# Diffuse Thyroid Lipomatosis and Amyloid Goiter With Incidental Papillary Thyroid Carcinoma: A Rare Case Report

**DOI:** 10.7759/cureus.57896

**Published:** 2024-04-09

**Authors:** Dona Maria George, Saloni Naresh Shah

**Affiliations:** 1 Department of Histopathology and Cytology, Apollo Hospitals, Chennai, IND

**Keywords:** amyloid goiter, congo red, papillary carcinoma thyroid, fat-containing lesions of the thyroid, thyroid lipomatosis

## Abstract

Lipoid lesions of the thyroid gland are very rare. Fat-containing thyroid lesions include a variety of clinical-pathological disorders, such as adenolipomas, thyrolipomatosis, and lipomatous tissue, in the event of amyloidosis. Herein, we report a case of diffuse thyrolipomatosis with amyloidosis and incidentally detected papillary carcinoma of the thyroid in a 51-year-old female patient who clinically presented with a multinodular goiter. Amyloidosis in papillary carcinoma of the thyroid is very rare and can be primary or secondary amyloidosis. Thyrolipomatosis, amyloid goiter, and papillary carcinoma of the thyroid is a rare combination, and to our knowledge, this is the third reported case in the literature. The association of amyloidosis and the rare occurrence of a differentiated carcinoma have to be considered, as in the case of thyroid lipomatosis.

## Introduction

Fat-containing thyroid gland tumors are relatively rare and typically consist of clinical-pathological disorders, including adenolipomas, thyrolipomatosis, and lipomatous tissue in the setting of amyloidosis [1]. An amyloid goiter is an extremely rare disorder characterized by the enlargement of the thyroid gland compounded by amyloid deposition [1-2]. An amyloid goiter could be due to primary or secondary amyloidosis [3]. Primary amyloidosis is generally associated with plasma cell dyscrasias, and secondary amyloidosis in the thyroid gland can be due to chronic inflammatory conditions, such as tuberculosis (TB), ulcerative colitis, Crohn's disease, chronic kidney disease, cystic fibrosis, or rheumatoid arthritis, and familial Mediterranean fever [4]. Differentiated carcinomas are quite rare within this kind of lesion. Diffuse lipomatosis of the thyroid gland, amyloid goiter, and papillary carcinoma of the thyroid is a rare combination, and only two cases are found in the literature to the best of our knowledge [1-2]. Here, we report the case of a 51-year-old female with diffuse thyroid lipomatosis, amyloid goiter, and incidentally detected papillary thyroid carcinoma.

## Case presentation

**Figure 1:**
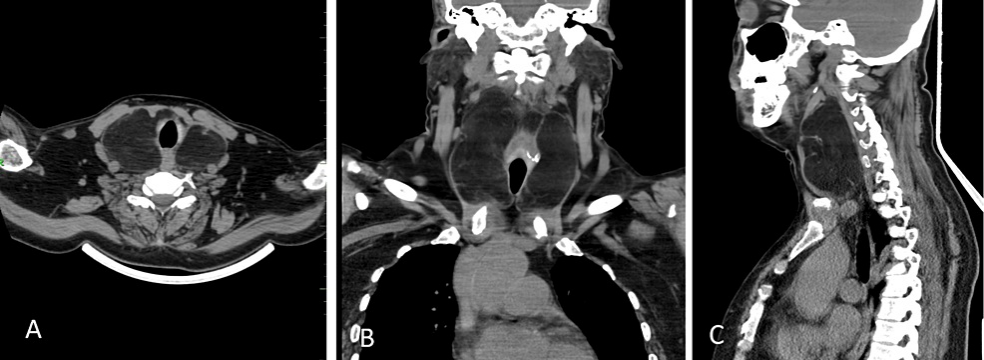
MRI findings A, B, and C: MRI of the neck showing a homogenously low-density mass of the neck with retrosternal and hypopharyngeal extension and bilateral displacement of the carotid sheath.

A 51-year-old female presented with a multinodular goiter for four years. The patient underwent radioiodine treatment for toxic multinodular goiter two years back, following which she was on replacement therapy for thyroid hormone. She has been a known case of stage 4 chronic kidney disease and amyloid nephropathy for the past three years. The patient was evaluated, and an MRI of the neck showed a homogenously low-density mass of the neck with retrosternal and hypopharyngeal extension with bilateral displacement of the carotid sheath (Figure 1).

**Figure 2:**
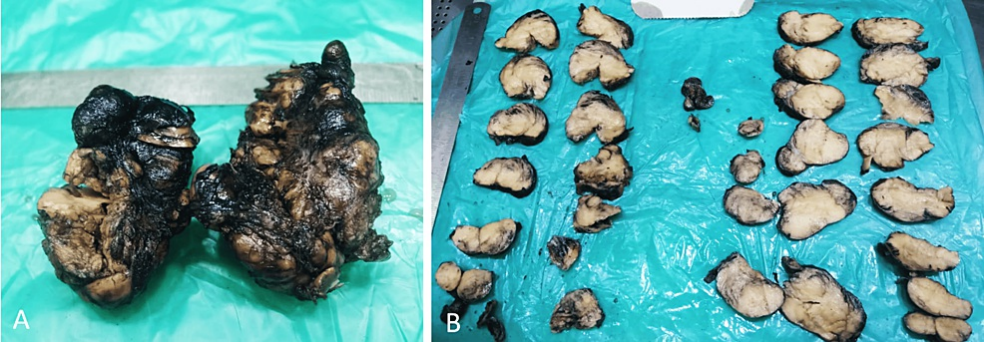
Macroscopic findings A and B: Enlarged thyroid gland with diffuse yellowish cut surface of the tumor macroscopically

The patient underwent a total thyroidectomy. Histopathological examination was done, and macroscopy showed an enlarged right lobe measuring 9.4 x 6.6 x 5.7 cm, an enlarged left lobe measuring 8.5 x 5.2 x 4.7 cm, and an isthmus measuring 2.2 x 1.2 x 1 cm. The cut surface of both lobes and isthmus was soft and diffusely yellowish with focal grey-white and reddish-brown congested areas (Figure 2). Microscopy showed lobules of adipocytes of varying sizes with thin-walled capillary-sized blood vessels interspersed with thick and thin fibrous septae. Areas of thyroid parenchyma with atrophic thyroid follicles are seen within the adipose tissue, with patchy foci of amorphous pink material, which polarizes with apple-green birefringence on Congo red stain.

**Figure 3:**
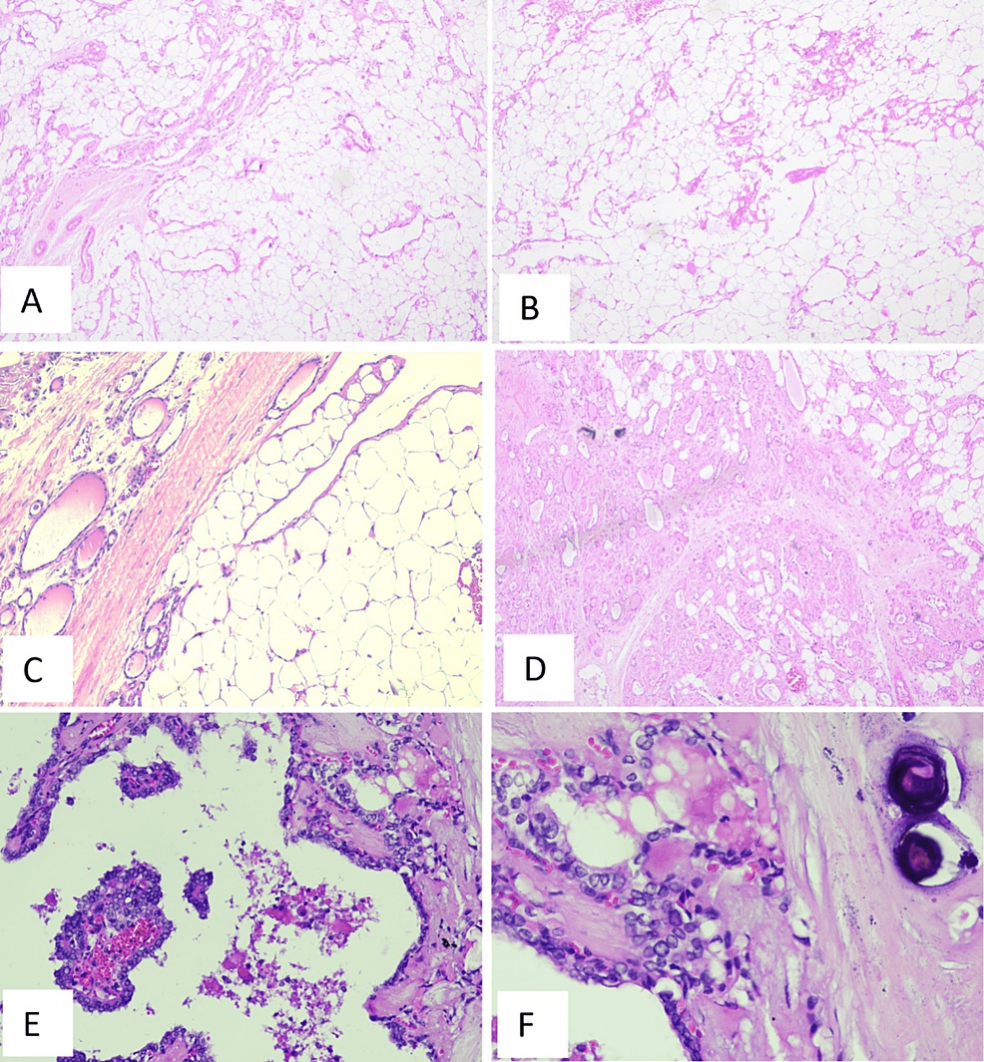
Light microscopy findings A and B: Light microscopy showing lobules of adipocytes of varying sizes with thin-walled capillary-sized blood vessels interspersed with thick and thin fibrous septae (H&E: 40X). C and D: Areas of thyroid parenchyma with atrophic thyroid follicles are seen within the adipose tissue (H&E: C-100X and D-40X). E and F: Papillary carcinoma of thyroid with papillary cores, nuclear grooving, nuclear overlapping, intranuclear cytoplasmic inclusions, and psammommatous calcifications (H&E: 400X).

**Figure 4:**
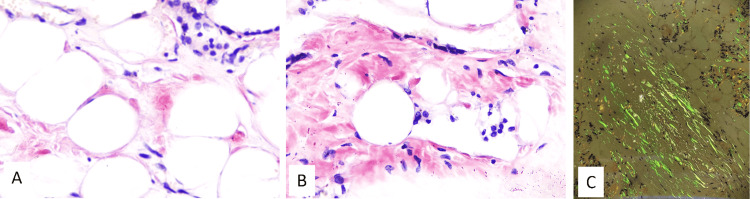
Special stains A and B: Light microscopy of the Congo red staining shows areas of amyloid in salmon color (Congo red: 400X). C: Polarized light microscopy of the Congo red staining shows apple-green birefringence in polarized light (100X).

Further tissue sections showed an incidental papillary carcinoma in two sections with papillary carcinoma of the thyroid with papillary cores, nuclear grooving, nuclear overlapping, intranuclear cytoplasmic inclusions, and psammommatous calcifications (Figures 3, 4). With all these features and further references, a diagnosis of papillary thyroid carcinoma with an amyloid goiter and thyroid lipomatosis was made.

The post-operative period was uneventful. The postoperative biochemical evaluation showed decreased serum-free T3 and T4, elevated TSH, and serum creatinine of 2.8 mg per deciliter. The patient was discharged from the hospital and disease-free on the six-month follow-up.

## Discussion

The thyroid gland is an unusual site for mature fat tissue and can be located in the perivascular region [3]. Only a few fat-containing thyroid lesions have been reported to date. Neoplastic lesions of the thyroid with fat are thyrolipoma, papillary carcinoma, and follicular carcinoma, while non-neoplastic lesions with an amyloid goiter are thyrolipomatosis, adenomatous nodule, dyshormonogenetic goiter, and lymphocytic thyroiditis [4]. Thyrolipomatosis is the most prevalent disorder in which mature fat cells infiltrate the normal thyroid gland. It typically has a uniform yellow cut surface resembling a benign lipomatous lesion with no obvious papillary architecture. No fibrous capsule development has been described, and stromal fibrosis and lymphocytic infiltration are occasionally detected [1]. Foci of adipose metaplasia can be seen in a multinodular goiter [5].

An amyloid goiter has been linked to widespread fatty proliferation, most likely as a result of tissue hypoxia caused by increasing capillary and thyroid follicle loss, which leads to stromal fibroblast metaplasia. There will be diffuse enlargement of the thyroid gland due to amyloidosis clinically presenting as a nodular goiter. Amyloid stromal deposition is common in medullary thyroid carcinoma, and it has been proposed that tumor cells can produce it specifically when significant amounts of calcitonin aggregate into insoluble fibrils. An amyloid stromal deposition is frequent in medullary thyroid carcinoma, and it has been proposed that tumor cells can produce it specifically when significant amounts of calcitonin aggregate into insoluble fibrils [3]. Chesky et al. observed in 1953 that this could manifest as congenital enlargement of the thyroid gland [6]. Several explanations have been offered to explain thyroid involvement by adipose tissue. Gnepp et al. suggested it as a congenital malformation [7]. Other researchers match the hypothesis of an event involving the insertion of adipose nests during development [8-9]. As indicated in this case report, an amyloid goiter can be linked with widespread fatty proliferation, most likely due to tissue hypoxia related to increasing capillary and thyroid follicle loss, resulting in stromal fibroblast metaplasia [10-11].

Patients will be mostly euthyroid with a nodular goiter. Pressure symptoms, such as dysphagia, dyspnea, and dysphonia, will be loud in most patients due to the large size and retrosternal extension. Imaging studies will not help in identifying an amyloid goiter. Ultrasound examination may show high echogenicity and very fine homogenous echotexture. Imaging results might vary depending on the quantity of amyloid and fat. Hyperechogenicity on ultrasound and low density on CT, as shown in our instance, may indicate fat tissue. 

The mainstay of diagnosis is fine-needle aspiration cytology (FNAC) and tissue biopsy, followed by special staining, polarizing microscopy, and immunohistochemistry. FNAC will show dense amorphous clumps of extracellular material and or irregularly shaped fragments with scalloped and pointed edges. The amorphous fragments stain eosinophilic on the Papanicolaou stain, magenta color on the Giemsa stain, and deep blue color with Diff-Quick stain. Histology sections show amorphous eosinophilic fibrillary material in perifollicular and perivascular regions and may replace the thyroid follicles. Congo red shows the salmon color and apple-green birefringence on polarized light. Immunohistochemical tests can aid in determining the origin of amyloid protein deposits, as primary amyloidosis contains light chains and serum amyloid A protein in secondary amyloidosis.

Papillary thyroid carcinoma and amyloid goiter have seldom been accompanied together, with just a few cases documented. The mechanism is unexplained [12-13]. Liftin et al. described a case of mixed papillary and follicular thyroid cancer accompanied by an amyloid goiter in a rheumatoid arthritis patient [14]. Coli et al. described a case of an amyloid goiter with diffuse lipomatosis and papillary thyroid carcinoma [2]. Papillary thyroid carcinoma within an amyloid goiter was reported by Coca-Pelaz et al. and Nessim et al. [15]. These cases had no lipomatosis being reported. 

We emphasize the need to incorporate diffuse lipomatosis and amyloid goiter in the differential diagnosis of a nodular goiter and the obligation to rule out differentiated thyroid carcinoma within an amyloid goiter. The above-discussed clinical and radiological features should always raise suspicion of diffuse thyrolipomatosis and associated malignancies.

## Conclusions

Fat-containing lesions of the thyroid gland are very rare. Thorough clinicoradiological correlation and pathological examinations will lead to the diagnosis of these rare entities. Various neoplastic and non‑neoplastic thyroid lesions can be associated with fatty infiltration of the thyroid gland. Therefore, the above-discussed lesions must be considered in the differential diagnosis of thyrolipomatosis. The association of amyloidosis and the rare occurrence of a differentiated carcinoma have to be considered, as in this case, so that conscious tissue sampling is done for accurate diagnosis and further proper treatment.
